# Microglial activation, tau and amyloid deposition in TREM2 p.R47H carriers and mild cognitive impairment patients: a multi-modal/multi-tracer PET/MRI imaging study with influenza vaccine immune challenge

**DOI:** 10.1186/s12974-023-02945-0

**Published:** 2023-11-21

**Authors:** Oliver Cousins, Julia J. Schubert, Avinash Chandra, Mattia Veronese, Polena Valkimadi, Byron Creese, Zunera Khan, Ryan Arathimos, Adam Hampshire, Ivana Rosenzweig, Clive Ballard, Anne Corbett, Dag Aasland, Latha Velayudhan, Michael O’Neill, David Collier, Ramla Awais, Kerstin Sander, Erik Årstad, Oliver Howes, Federico Turkheimer, Angela Hodges

**Affiliations:** 1 Institute of Psychiatry, Psychology and Neuroscience, King’s College London, London, SE5 9RT UK; 2 Department of Information Engineering, University of Padua, 35131 Padua, Italy; 3 College of Medicine and Health, University of Exeter, Exeter, EX1 2HZ UK; 4 Faculty of Medicine, Imperial College London, London, SW7 2AZ UK; 5 Eli Lilly and Company, Basingstoke, RG21 4FA UK; 6 Centre for Radiopharmaceutical Chemistry, University College London, London, WC1E 6BS UK; 7 Division of Psychology, Department of Life Sciences, Brunel University London, London, UB8 3PH UK

**Keywords:** *TREM2*, Neuroinflammation, TSPO, Florbetapir, AV1451, DPA714, Alzheimer’s disease, Microglia, PET

## Abstract

**Background:**

Microglia are increasingly understood to play an important role in the pathogenesis of Alzheimer’s disease. The rs75932628 (p.R47H) *TREM2* variant is a well-established risk factor for Alzheimer’s disease. TREM2 is a microglial cell surface receptor. In this multi-modal/multi-tracer PET/MRI study we investigated the effect of *TREM2* p.R47H carrier status on microglial activation, tau and amyloid deposition, brain structure and cognitive profile.

**Methods:**

We compared *TREM2* p.R47H carriers (*n* = 8; median age = 62.3) and participants with mild cognitive impairment (*n* = 8; median age = 70.7). Participants underwent two [^18^F]DPA-714 PET/MRI scans to assess TSPO signal, indicative of microglial activation, before and after receiving the seasonal influenza vaccination, which was used as an immune stimulant. Participants also underwent [^18^F]florbetapir and [^18^F]AV1451 PET scans to assess amyloid and tau burden, respectively. Regional tau and TSPO signal were calculated for regions of interest linked to Braak stage. An additional comparison imaging healthy control group (*n* = 8; median age = 45.5) had a single [^18^F]DPA-714 PET/MRI. An expanded group of participants underwent neuropsychological testing, to determine if *TREM2* status influenced clinical phenotype.

**Results:**

Compared to participants with mild cognitive impairment, *TREM2* carriers had lower TSPO signal in Braak II (*P* = 0.04) and Braak III (*P* = 0.046) regions, despite having a similar burden of tau and amyloid. There were trends to suggest reduced microglial activation following influenza vaccine in *TREM2* carriers. Tau deposition in the Braak VI region was higher in *TREM2* carriers (*P* = 0.04). Furthermore, compared to healthy controls *TREM2* carriers had smaller caudate (*P* = 0.02), total brain (*P* = 0.049) and white matter volumes (*P* = 0.02); and neuropsychological assessment revealed worse ADAS-Cog13 (*P* = 0.03) and Delayed Matching to Sample (*P* = 0.007) scores.

**Conclusions:**

*TREM2* p.R47H carriers had reduced levels of microglial activation in brain regions affected early in the Alzheimer’s disease course and differences in brain structure and cognition. Changes in microglial response may underlie the increased Alzheimer’s disease risk in *TREM2* p.R47H carriers. Future therapeutic agents in Alzheimer’s disease should aim to enhance protective microglial actions.

**Supplementary Information:**

The online version contains supplementary material available at 10.1186/s12974-023-02945-0.

## Introduction

The classical neuropathological hallmarks of Alzheimer’s disease are abnormal amyloid-beta (Aβ) and tau protein aggregates, and neurodegeneration. Microglia are the resident immune cell of the brain and their importance in the pathogenesis of Alzheimer’s disease is increasingly recognised [1]. Advances in genetic research have revealed that microglia associated genes account for approximately 25% of Alzheimer’s disease risk genes [2, 3]. In Alzheimer’s disease, activated microglia cluster around amyloid plaques where they potentially act as a barrier mitigating the neurotoxic effects of Aβ [4]. They may also clear Aβ via phagocytosis [5]. Tau pathology initially accumulates in transentorhinal regions before propagation to limbic then wider neocortical regions and is highly correlated with neuronal cell loss and the emergence and progression of symptoms in patients [6]. This progressive spread has been divided into six Braak stages, based on histopathological studies [7]. Recently microglia were shown to internalise tau aggregates [8] and in so doing, appear to contribute to the spread of tau pathology [9–11].

One of the highest impact risk gene variants for Alzheimer’s disease is in the gene for *TREM2* (triggering receptor expressed on myeloid cells 2). The rare *TREM2* rs75932628 non-synonymous coding variant (p.R47H) has a similar effect size to APOEε4, with a 2- to 3-fold increased risk of Alzheimer’s disease in heterozygous carriers [12, 13]. TREM2 is a microglia cell surface receptor, which promotes a change in microglial phenotype and phagocytosis following the binding of ligands, including lipid species, APOE and Aβ [14–17]. Evidence suggests the *TREM2* p.R47H variant leads to impaired ligand binding [18]. *TREM2* p.R47H may therefore act to increase the risk of Alzheimer’s disease via a partial loss of a protective function of microglia [19].

There have been a limited number of studies examining the clinical and pathological characteristics of *TREM2* p.R47H carriers. One study reported a higher proportion of psychiatric and parkinsonian symptoms in *TREM2* p.R47H carriers who received an Alzheimer’s disease diagnosis [20], while others failed to find any distinguishing clinical symptoms [21, 22]. Smaller hippocampal volumes in older, but cognitively normal, carriers have also been reported [23]. Amyloid burden detected via PET scan in people with Alzheimer’s disease was not found to differ between carriers and non-carriers of the *TREM2* p.R47H variant [24]. However, recent preclinical research has shown that TREM2 acts to reduce tau seeding in the presence of significant Aβ pathology [25].

TSPO (translocator protein) is a mitochondrial membrane protein with an uncertain physiological role. It is usually expressed at low levels in the brain [26]. However, TSPO protein expression is upregulated in response to a variety of insults, including immune challenges, and is a marker of microglial activation when examined at post-mortem [27]. We interpret the increase in TSPO signal broadly as ‘microglial activation’ in this paper. However, it is important to recognise that microglia are now understood to have a diverse array of phenotypes beyond the traditionally recognised ‘resting’ and ‘activated’ states [28], which are not measurable using in vivo TSPO-PET. We measured TSPO signal using the second-generation TSPO-PET tracer [^18^F]DPA-714, which has a good signal to noise ratio [29]. We used the seasonal influenza vaccine as an immune challenge, which in mice has been shown to increase microglial activation [30, 31]. Abnormal protein aggregation was also measured using [^18^F]florbetapir for amyloid and [^18^F]AV1451 (flortaucipir) for tau.

The primary aim of this study was to investigate if *TREM2* p.R47H risk variant carriers have reduced in vivo microglial activation, measured using TSPO signal, compared to non-carriers also at increased risk of Alzheimer’s disease. We assessed microglia activation at baseline and following an immune stimulant. Additional aims were to establish if the deposition of amyloid or tau differs between *TREM2* p.R47H carriers and non-carriers, and simultaneously explore the relationship between amyloid burden, tau burden and microglial activation in these cases. We also investigated whether there were differences in brain structure and cognitive profiles that distinguished *TREM2* p.R47H carriers from non-carriers.

## Materials and methods

### Participants

Participants were recruited from existing research cohorts established at King’s College London, including the Alzheimer’s disease research cohorts AddNeuroMed and KHP-DCR (King’s Health Partners—Dementia Case Register) [32], and PROTECT (Platform for Research Online to investigate Genetics and Cognition in Aging—REC reference 13/LO/1578), a cohort of healthy older adults. All studies had consent for re-contact for future research studies [33]. AddNeuroMed, KHP-DCR and PROTECT are longitudinal studies involving annual cognitive assessments. Imputed whole genome data (Human610-Quad genotyping platform, AddNeuroMed and KHP-DCR; Illumina Global Screening Array with custom content, PROTECT) were used by the cohort managers to invite a subset of cases heterozygous for the rare *TREM2* p.R47H risk variant or homozygous for the common non-risk variant, to participate in PHAGO. Genotypes linked to individuals were unknown to the PHAGO study team at recruitment but were later established by sequencing exon 2 of *TREM2* using DNA extracted from blood and/or saliva. Additionally, participants with mild cognitive impairment were recruited from memory clinics within the South London and the Maudsley Hospital Trust and the Join Dementia Research online platform.

General inclusion criteria for assessment were (i) 50–80 years old and (ii) able to give informed consent. Exclusion criteria were (i) history of significant neurological or psychiatric disorders and (ii) current or recent history of drug or alcohol abuse. Only participants found to be high-affinity (HAB) or mixed-affinity (MAB) binding for the TSPO polymorphism rs6971 underwent imaging, as low-affinity binders show negligible TSPO-PET signal [26]. Additional exclusion criteria for imaging assessments were (i) contraindications to the seasonal flu vaccine, (ii) pregnancy or breastfeeding, (iii) contraindication to MRI, (iv) history of cancer within the last 5 years, (v) systemic steroid therapy. Participants with mild cognitive impairment had (i) a subjective memory complaint, (ii) objective cognitive impairment measured on neuropsychological testing (1.5 standard deviations below control mean), (iii) Clinical Dementia Rating (CDR) of 0.5 [34] and, (iv) preserved activities of daily living. Participants not eligible for imaging assessments following clinical and genetic screening were included only for the clinical and neuropsychological assessments.

Additionally, healthy control data for TSPO-PET and MRI were obtained from prior studies, using the same PET scanner and protocol, to enable normative comparisons of baseline (pre-vaccine challenge) TSPO levels and brain structure. These control participants met the general inclusion and exclusion criteria described above, except participants aged under 50 were also included. They were only genotyped for their TSPO binding status.

### Study activities

Participants underwent an initial screening visit involving assessment of medical history and physical examination. Detailed clinical assessments included the Geriatric Depression Scale (GDS) [35], Hamilton Anxiety Rating Scale (HAM-A) [36], Apathy Evaluation Score (AES) [37], Quality of life in Alzheimer’s disease (QoL-AD) [38] and fatigue severity score [39]. Neuropsychological assessments included the Montreal Cognitive Assessment (MoCA) [40], FAS and animal naming fluency tasks [41], Trail-Making Task (TMT) [42], ADAS-Cog 13 [43] and a CANTAB computerised battery (Reaction Time [RTI], Paired Associates Learning [PAL], Spatial Working Memory [SWM], Delayed Matching to Sample [DMS], Rapid Visual Information Processing [RVP], Spatial Span [SSP], Pattern Recognition Memory [PRM] and One Touch Stockings of Cambridge [OTS]) [44]. Eligible participants underwent imaging assessments.

### Genotyping

Blood was collected in a 3 ml EDTA Vacuette or alternatively, saliva was provided by participants in a Genefix Saliva DNA/RNA collection and stabilisation tube (GFX-02, Isohelix), where blood collection was not practical. DNA was isolated using standard protocols followed by PCR and Sanger sequencing to establish the genotypes of the following variants: *TREM2* rs75932628 (p.R47H), TSPO rs6971 and APOE rs429358 and rs7412 (to derive APOE haplotypes ε2, 3 or 4).

### PET and MRI imaging

Participants underwent MRI on the 3 T SIEMENS Biograph mMR, a combined PET–MR machine. Scans took place at the King's College London & Guy's and St Thomas' PET Centre, London. A T1 weighted MPRAGE (magnetisation prepared rapid gradient echo) sequence with 1 mm^3^ voxel size was obtained (repetition time = 2300 ms, echo time = 2.96 ms, flip angle of 9). Images from the baseline scan were processed using FreeSurfer version 6.0 [45]. The DKT (Desikan–Killiany–Tourville) and ASEG (automated subcortical segmentation) atlases [46, 47] were used to obtain volumes of the following regions of interest (ROI): total brain (sum of grey matter and white matter), white matter, hippocampus, putamen and caudate, and frontal, temporal, and parietal grey matter. Intracranial volume measurements were also obtained as this can influence regional volumes [48]. The volume of T1 hypointensities was also obtained from FreeSurfer as a measure of leukoaraiosis [49]. Manual quality control of FreeSurfer output was undertaken as per the software manual—https://surfer.nmr.mgh.harvard.edu/fswiki/FsTutorial/TroubleshootingData.

TSPO and tau uptake within grey matter was compared within FreeSurfer-derived ROIs mapped to the six Braak stages of tau deposition in Alzheimer’s disease, further details of the regions used are available elsewhere [50]. It should be noted that the tau signal in the Braak II region may be affected by off-target binding to the choroid plexus, so results in this area should be interpreted with caution [51]. The tau Braak regions have been used to evaluate TSPO uptake in a prior study [9].

[^18^F]DPA-714 scans were performed before and 7 days after the influenza vaccine. Three *TREM2* p.R47H carriers and two participants with mild cognitive impairment did not undergo repeat imaging due to tracer supply issues. A mean dose of 184.3 (± 14.8) MBq was injected. Dynamic data were collected over 60 min and binned in 26 frames (1 × 60, 8 × 15, 3 × 60, 5 × 120, 9 × 300). Scans took place on a SIEMENS Biograph mMR PET/MRI in the afternoon at the King's College London & Guy's and St Thomas' PET Centre, London. Participants also underwent a CT head scan, which was used for attenuation correction [52]. Distribution volume ratio (DVR) values for ROIs were calculated using a simplified reference tissue model accounting for vascular tracer activity [53–55] and a supervised reference region approach, which has previously been validated for use with [^18^F]DPA-714 [56]. The method employed to derive the image-derived input function used to account for vascular binding was adapted from a previous study [53]. The blood pool was defined by selecting the 50 voxels with the highest activity during the initial 1.5 min of the dynamic PET scan, before the signal peak. The supervised reference region was determined using a set of pre-defined kinetic classes to identify cerebellar grey matter voxels with kinetic behaviour most similar to healthy grey matter. Partial volume effects were investigated by rerunning the analysis with partial volume correction (PVC) applied to each dynamic PET frame using the PETPVC toolbox [57].

The mean injected dose for the [^18^F]AV1451 scan was 180.3 (± 1.7) MBq. Participants had an 80-min uptake time followed by a 30-min dynamic scan. Scans took place on a Siemens Biograph™ TruePoint™ PET/CT at the Invicro centre for imaging sciences, London. Standardised uptake value ratio (SUVR) values were created by dividing the activity averaged over ROI voxels by the activity averaged over cerebellar grey matter voxels [58].

The mean injected dose for the [^18^F]florbetapir scan was 192.2 (± 45.4) MBq. Participants had a 40-min uptake time followed by a 20-min static scan. Scans took place on a GE Discovery PET/CT 710 at the Department of Nuclear Medicine, King’s College Hospital, London*.* One participant had a delayed scan start, 84 min following injection, due to scanner malfunction. At this time point the activity of [^18^F]florbetapir is expected to be sufficiently stable to allow for the SUVR analysis [59], so the data were included. To determine amyloid positivity a cortical summary region was created, comprising the FreeSurfer grey matter frontal, cingulate, lateral parietal, and lateral temporal regions. This value was divided by the signal within the whole cerebellum and a cut of 1.11 was applied, as per prior studies [60].

PET and MRI images were pre-processed using MIAKAT™ software, which allows for step-by-step quality control checks [61]. MPRAGE MRI underwent brain extraction and segmentation. Dynamic [^18^F]DPA-714 and [^18^F]AV1451 PET were corrected for motion and all PET images were co-registered with baseline MPRAGE MRI. ROI maps were defined based on the individual participant FreeSurfer template. The CIC (Clinical Imaging Centre) v2.0 neuroanatomical atlas [62] was non-linearly transformed to baseline MPRAGE MRI. The reference regions used for TSPO and tau analyses were defined using a combination of grey matter segmentation output and the transformed CIC atlas. Time activity curves were then extracted from the pre-processed PET images.

### Influenza vaccine challenge

Following the first TSPO-PET scan participants were given the cell-based quadrivalent influenza vaccine, Flucelvax Tetra™, based on the 2019/2020, 2020/2021 and 2021/2022 composition. This was consistent with established clinical practice in older eligible participants as part of seasonal health protection measures. Scans were scheduled to coincide with participants' planned vaccination or were delayed until after the winter flu season, if already vaccinated. Blood samples were collected before and 4–10 weeks after influenza vaccination to establish seropositivity. Serum was isolated and stored at −80 °C, prior to being sent to Public Health England for the evaluation of pre- and post-vaccination antibody levels against the 2020/21 influenza strains, using a haemagglutination inhibition assay (HAI). A HAI titre of 40 of more was indicative of seroconversion to a protective antibody response [63].

### Statistical analysis

The *TREM2* p.R47H carrier group was compared to the mild cognitive impairment group as both were at higher risk of Alzheimer’s pathology. *TREM2* p.R47H carriers were also compared against a healthy control group (imaging control group) for the TSPO and MRI imaging assessments, and against a separate healthy control group (clinical control group) for clinical measures. Demographic variables were compared between comparison groups using the Mann–Whitney *U* test for continuous variables (as distribution not normal) and Chi-squared or Fisher’s exact test for categorical variables depending on participant number.

A general linear model was also used to assess differences in levels of tau and baseline TSPO across Braak-defined regions of interest, with respect to study group, with TSPO status (for TSPO results) and age as covariates, as increased TSPO signal is observed in HABs vs MABs [26] and with increasing age [64]. For the response to influenza vaccination, a linear mixed model was used to assess for an interaction between study group and change in TSPO signal pre- and post-vaccination across the Braak regions. Age and TSPO genetic status were used as covariates. Participant ID was used as a random factor, and random intercept and slope were included to account for between participant variation. Linear regression was used to assess the association between TSPO regional activity and tau deposition (Braak I), amyloid positivity, age and TSPO status. Significant results for TSPO related outcomes (our primary aims) underwent Bonferroni multiple comparison correction to account for 6 tests. Brain structure volumes of interest extracted from structural MRI were compared using a general linear model with age, sex and intracranial volume as covariates.

Clinical assessment scores were compared using the Mann–Whitney *U* test (non-normal distribution) or t-test (normal distribution). Neuropsychological assessment scores were compared using a general linear model with age and years of education as covariates. Positive skew was corrected for by Log10 transformation for TMT-A, TMT-B, ADAS-Cog 13, OTS mean choice to correct, OTS mean latency to correct, PAL (total errors adjusted), PAL (total errors 6 shapes), RTI 5 choice reaction and RVP mean latency. Normality of residuals for the general linear model and linear regression were established by inspection of the histograms and Q–Q plots. All statistics were carried out in SPSS version 27.

## Results

### Demographics

Eight *TREM2* p.R47H carriers underwent PET and MRI assessments. Demographic characteristics were compared against eight participants with mild cognitive impairment and eight imaging controls (Table 1). The mild cognitive impairment group was older than the *TREM2* p.R47H group (70.7 vs 62.3; *P* = 0.01). Imaging controls were younger (45.5 vs 62.3; *P* < 0.001) and contained a higher proportion of men than the *TREM2* p.R47H group (100% vs 62.5%; *P* = 0.03). For the subgroup of participants undergoing repeat TSPO-PET scans, the mild cognitive impairment group was older than the *TREM2* p.R47H group (70.7 vs 61.7; *P* = 0.03; Additional file 1: Table S1).

**Table 1:** Demographic characteristics of participants undergoing MRI and PET

	Imaging controls (*n* = 8)	MCI (*n* = 8)	*TREM2* p.R47H (*n* = 8)
Age (years) (median + IQR)	45.5 (43.3–48.8)	70.7 (64.0–75.8)	62.3 (60.7–67.7)^a,b^
Sex (male/female)	8/0	6/2	5/3^a^
TSPO genotype (MAB/HAB)	2/6	5/3	4/4
APOEε4 (carrier/non-carrier)	–	4/4	2/6
Amyloid status (positive/negative)	–	2/6	1/7
WM—hypointensity volume (cm^3^) (median + IQR)	1.0 (0.9–1.6)	1.9 (1.4–4.6)	1.5 (0.9–2.0)

APOEε4 carrier refers to the number with ≥ 1 ε4 allele

Positive amyloid status refers to having a summary cortical SUVR of > 1.11 on amyloid PET

*HAB* high-affinity binder, *IQR*  interquartile range, *MAB*  mixed-affinity binder, *MCI*  mild cognitive impairment, *TSPO*  translocator protein, *WM*  white matter

^a^*TREM2* p.R47H carrier significant versus controls; *P* < 0.05

^b^*TREM2* p.R47H carrier significant versus MCI group; *P* < 0.05

For the clinical and neuropsychological assessments, *TREM2* p.R47H carriers were compared to a mild cognitive impairment and a healthy control group (clinical controls) (Table 2). The clinical control group were a different group of people from the imaging control group and had a similar age to the mild cognitive impairment group. *TREM2* p.R47H carriers were slightly younger than the mild cognitive impairment group (64.9 vs 71.6; *P* = 0.02) and clinical control group (64.9 vs 73.1; *P* = 0.005). The mild cognitive impairment group had worse MoCA scores (25 vs 29; *P* = 0.003) and fewer years of education (14 vs 18; *P* = 0.01) compared to *TREM2* p.R47H carriers. As would be anticipated, given their diagnosis, the mild cognitive impairment group had a worse MoCA score than the clinical control group (25 vs 28;* P* < 0.001).

**Table 2:** Demographic characteristics of participants undergoing clinical and neuropsychological assessments

	Clinical controls (*n* = 29)	MCI (*n* = 11)	*TREM2* p.R47H (*n* = 12)
Age (median + IQR)	73.1 (66.5–76.4)	71.6 (65.8–77.5)	64.9 (60.0–69.4)^a,b^
Sex (male/female)	10 / 19	8 / 3	6 / 6
Education (median + IQR)	18 (16.5–20)	14 (12–18)	18 (17.3–20)^b^
APOEε4 (carrier/non-carrier)	8 / 21	4 / 7	2 /10
MoCA (median + IQR)	28.0 (27.0–30.0)	25.0 (23.0–26.0)	29.0 (26.5–30.0)^b^

APOEε4 carrier refers to the number with ≥ 1 ε4 allele

*IQR*  interquartile range, *MCI*  mild cognitive impairment, *MoCA*  Montreal Cognitive Assessment

^a^*TREM2* p.R47H carrier significant versus controls; *P* < 0.05

^b^*TREM2* p.R47H carrier significant versus MCI group; *P* < 0.05

### Microglial activation

**Fig. 1:**
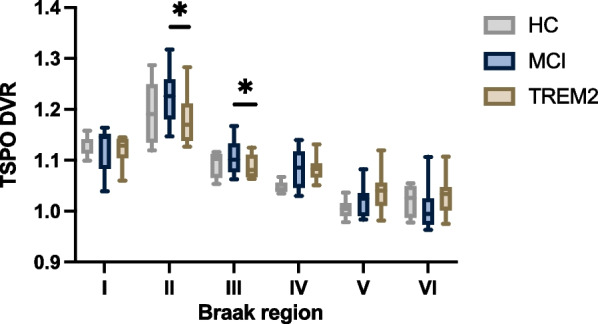
Baseline comparison of regional microglial activation. TSPO DVR values across Braak stages, comparing HC, MCI and *TREM2* p.R47H carriers, showing lower DVR in Braak II and Braak III regions in *TREM2* p.R47H carriers. Box plots show median values, interquartile range and range. Statistical comparisons between the *TREM2* p.R47H group and each of the control groups separately, with age and TSPO status as covariates. * *P* < 0.05. *DVR*  distribution volume ratio, *HC*  health control, *MCI*  mild cognitive impairment, *TSPO*  translocator protein

We compared baseline differences in microglial activation between *TREM2* p.R47H carriers and both participants with mild cognitive impairment and an imaging healthy control group (Fig. 1 and Additional file 1: Table S2). Reduced TSPO signal was found in Braak II (*η*^2^ = 0.31; *F* = 5.41; *P* = 0.04) and III (*η*^2^ = 0.29; *F* = 4.95; *P* = 0.046) regions of interest between *TREM2* p.R47H carriers and mild cognitive impairment participants, which remained significant after PVC application (Braak II: *P* = 0.03; Braak III: *P* = 0.03). These significant results do not withstand multiple comparison correction. While TSPO signal in these regions was also lower in *TREM2* p.R47H carriers compared to imaging controls, the difference was not statistically significant. There were no differences in reference region SUV values between the groups.

### Microglial activation—influenza vaccine challenge

**Fig. 2:**
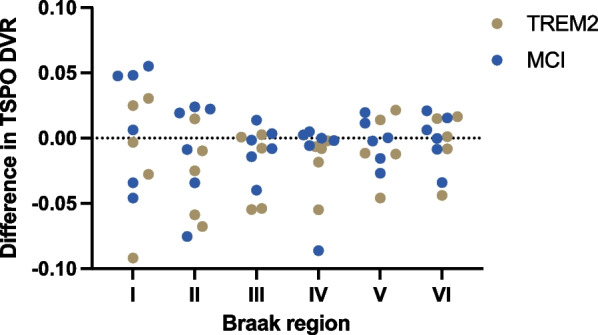
Microglia response to influenza vaccine. Change in TSPO DVR values in response to influenza vaccine are presented based on Braak regions and participant group. *DVR*  distribution volume ratio, *MCI*  mild cognitive impairment, *TSPO*  translocator protein

The influenza vaccine did not result in a significant change in TPSO signal in any of the Braak regions (Fig. 2 and Additional file 1: Table S3). However, within Braak II (*β* = − 0.03 ± 0.02; *P* = 0.08), Braak III (*β* = − 0.02 ± 0.01; *P* = 0.08), Braak IV (*β* = − 0.02 ± 0.01; *P* = 0.06) there were trends to suggest that influenza vaccine lowered the TSPO signal in *TREM2* p.R47H carriers compared to mild cognitive impairment participants.

Where pre- and post-vaccine serum was available, serological conversion was assessed for participants, undergoing repeat TSPO scans, who were given the 2020/21 influenza vaccine (*n* = 7 out of 11). Four of these participants had evidence of seroconversion, whereas three participants had protective levels of antibodies both pre- and post-vaccination. Three additional participants, who only had baseline TSPO scans, also had evidence of serological conversion.

### Amyloid and tau pathology

**Fig. 3:**
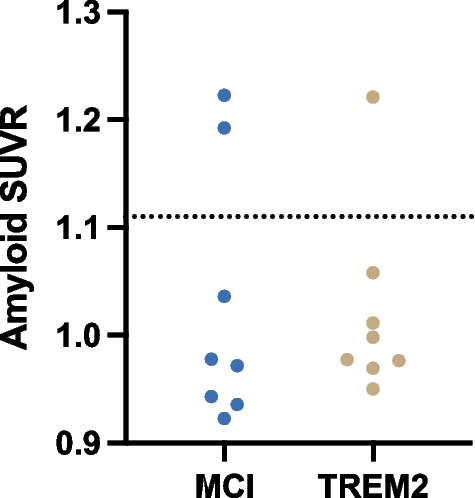
Amyloid burden. SUVR values for [^18^F]florbetapir between MCI and *TREM2* p.R47H carrier groups. Dotted line represents the threshold of 1.11 for amyloid positivity. *MCI*  mild cognitive impairment, *SUVR*  standardised uptake value ratio

One *TREM2* p.R47H carrier and two mild cognitive impairment participants reached the threshold for abnormal amyloid pathology (Fig. 3) [60]. Given low participant numbers, amyloid positive and negative participants were therefore pooled together for subsequent analyses.

**Fig. 4:**
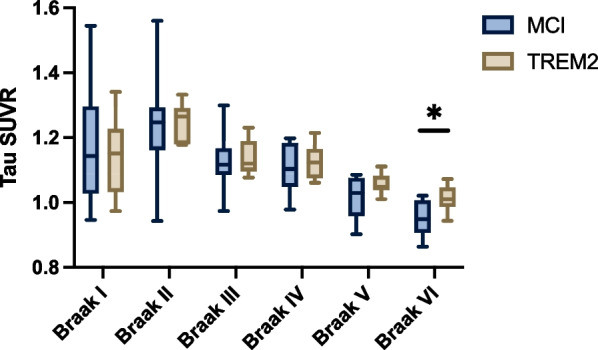
Tau burden. SUVR values for [^18^F]AV1451 across Braak stages, comparing MCI to *TREM2* p.R47H carrier groups. Box plots show median values, interquartile range and range. Statistical comparisons were made with age as a covariate. * *P* < 0.05. *MCI*  mild cognitive impairment, *SUVR*  standardised uptake value ratio

There was no difference in regional tau PET signal between *TREM2* p.R47H and mild cognitive impairment participants in Braak regions I–V regions of interest (Fig. 4, Additional file 1: Table S4). *TREM2* p.R47H carriers did however have higher tau PET signal in Braak VI than mild cognitive impairment participants (*η*^2^ = 0.28; *F* = 5.0; *P* = 0.04), although it should be noted that uptake values overall were low in this region in all participants, which is to be expected in the early-stage disease period we were focused on.

In order to confirm an anticipated association between amyloid positivity and early tau burden, a pooled analysis of all subjects was performed, demonstrating that amyloid positivity was associated with higher Braak I tau region of interest deposition (r_pb_ = 0.60; *P* = 0.01; Additional file 1: Fig. S1).

### Relationship between microglial activation, tau and amyloid pathology

We constructed linear regression models to assess if TSPO signal across the Braak stage regions of interest could be predicted by early tau burden (Braak I), amyloid positivity, age and TSPO binding status. There was no significant association in either the mild cognitive impairment group or *TREM2* p.R47H carriers.

### MRI

ROI volumes were compared between *TREM2* p.R47H carriers and, both control and mild cognitive impairment groups (Table 3). Total brain volume (*η*^2^ = 0.31; *F* = 4.86; *P* = 0.049), white matter volume (*η*^2^ = 0.42; *F* = 8.04; *P* = 0.02) and caudate volume (*η*^2^ = 0.41; *F* = 7.67; *P* = 0.02) were smaller in the *TREM2* p.R47H group compared to controls, with age, sex and intracranial volume included as covariates.

**Table 3:** FreeSurfer-derived regions of interest volumes (cm^3^) were compared based on study group

Region	Imaging controls (*n* = 8)	MCI (*n* = 8)	*TREM2* p.R47H (*n* = 8)
TBV (mean ± SD)	1244 ± 94.2	999.6 ± 107.6	1029 ± 47.0^a^
Frontal (mean ± SD)	175.0 ± 18.2	151.2 ± 14.2	156.5 ± 11.5
Temporal (mean ± SD)	118.1 ± 12.1	102.8 ± 12.2	99.56 ± 7.20
Parietal (mean ± SD)	114.3 ± 15.7	98.88 ± 10.3	104.1 ± 4.32
Hippocampus (mean ± SD)	9.191 ± 0.724	7.258 ± 0.740	7.826 ± 0.570
Putamen (mean ± SD)	10.77 ± 0.563	8.871 ± 1.33	9.467 ± 0.993
Caudate (mean ± SD)	7.526 ± 0.619	6.531 ± 0.944	6.438 ± 0.519^a^
White matter (mean ± SD)	559.5 ± 47.7	410.3 ± 63.4	424.8 ± 23.5^a^

Age, sex and intracranial volume included as covariates for statistical comparison

*MCI*  mild cognitive impairment, *SD*  standard deviation, *TBV*  total brain volume

^a^*TREM2* p.R47H carrier significant *versus* controls; *P* < 0.05

### Clinical and neuropsychological assessments

Medical history revealed that none of the *TREM2* p.R47H carriers were experiencing features of parkinsonism, hallucinations or delusions. A series of clinical assessment scales were used to determine if *TREM2* p.R47H carriers exhibited differences in depression, anxiety, apathy, fatigue and quality of life compared to the clinical control and mild cognitive impairment group, who did not have the risk variant (Additional file 1: Table S5). The mild cognitive impairment group had worse assessment scores than the *TREM2* p.R47H group, across all domains. There was no difference between the *TREM2* p.R47H and clinical control group.

Neuropsychological battery revealed that the *TREM2* p.R47H group had a worse performance on ADAS-Cog 13 (*F* = 5.3; *η*^2^ = 0.13; *P* = 0.03) and DMS (*F* = 8.3; *η*^2^ = 0.19; *P* = 0.007) when compared to the clinical control group, with age and years of education as covariates. These detailed neuropsychological assessments did not reveal a difference between the MCI and *TREM2* p.R47H groups except a possible trend for participants with mild cognitive impairment to have worse ADAS-Cog 13 scores than *TREM2* p.R47H carriers (*P* = 0.09).

## Discussion

Within this multi-modal PET/MRI imaging study we have shown that carriers of the rare *TREM2* p.R47H Alzheimer’s disease risk variant have lower levels of TSPO tracer uptake in brain regions known to be affected in early Alzheimer’s disease. This is consistent with our hypothesis that *TREM2* p.R47H carriers have reduced microglial activation and shows this for the first time in vivo in people. The influenza vaccine was not shown to be an effective stimulator of brain microglial activation, measured by TSPO-PET in any study group. We have additionally shown that older *TREM2* p.R47H carriers have subclinical impaired cognitive performance and areas of reduced brain volume compared to controls.

Lower relative TSPO uptake was specifically found in the hippocampus (included in Braak II staging) and medial/inferior temporal lobe regions (Braak III) in *TREM2* p.R47H carriers, although these results did not remain after stringently adjusting for multiple comparison testing. This is in keeping with preclinical studies which have shown lower levels of hippocampal microglial activation in mice carrying the human *TREM2* p.R47H variant, with or without Alzheimer’s disease pathology [65, 66]. The comparison group for this aspect of the study was a mild cognitive impairment group. Therefore, the comparison was between two groups at higher risk of Alzheimer’s disease, although the ultimate diagnostic outcome of participants was unknown. Additionally, no difference in tau deposition in these regions was found between the two groups and there was a similar number of amyloid positive cases, suggesting reduced microglial activation in *TREM2* p.R47H despite having a similar tau and amyloid burden to MCI cases. Abnormal amyloid and tau protein accumulation can pre-empt Alzheimer’s disease clinical symptoms by many years [67], and it has recently been hypothesised that tau, rather than amyloid, could be the initiating pathology [68]. The role of microglia in these initial stages of Alzheimer’s disease pathogenesis is thought to be protective, with microglia acting to reduce the spread of amyloid and possibly tau [19, 69]. It is possible that the expected loss of function in TREM2 conferred by p.R47H may underlie the lower levels of TSPO signal seen in the *TREM2* p.R47H carriers and that this reduced microglial activation may be a factor in the increased risk of Alzheimer’s disease in these carriers. This is consistent with in vitro work in iPSC derived microglia that shows TREM2 impairment leads to a locked immunometabolic block which prevents microglial activation in the presence of damage stimuli [70].

The influenza vaccine was utilised as an immune challenge based on animal models which demonstrated a brain microglial activation response [30, 31]. It was also chosen due to high levels of participant acceptability and familiarity, with millions of people receiving the vaccine each year in the UK [71]. Other immune challenges could have been considered including lipopolysaccharide, which has been shown to cause raised brain TSPO-PET signal [72]. However, the side effect profile makes this unacceptable for use in people [73]. The influenza vaccine was not shown to cause microglial activation, measured by TSPO signal, in this study. This was despite evidence of a peripheral antibody response in many of the participants. Another potential stimulant of brain microglia, interferon-alpha, has also recently failed to demonstrate TSPO signal change following administration [74]. The reason for this lack of microglial activation could potentially relate to a reduction in blood–brain-barrier permeability, and thus tracer transfer into the brain, in response to modest peripheral immune activation typically obtained with interferon-alpha and flu vaccines, while lipopolysaccharide-like stimulation is far more potent and may induce blood–brain-barrier leakage leading to higher tracer binding [75].

There was no difference in tau deposition between regions with different Braak stages comparing *TREM2* p.R47H carriers and non-carriers with mild cognitive impairment, except for higher deposition in the Braak stage VI region in *TREM2* p.R47H carriers. It is unclear whether this has biological relevance given that binding in this area was minimal across groups. However, it is notable that a higher early tau burden in this region is seen in the posterior cortical atrophy variant of Alzheimer’s disease [76]. This would be in keeping with a report that *TREM2* variant carriers are more likely to develop an atypical variant of Alzheimer’s disease [77]. There was an insufficient number of participants who were amyloid positive to meaningfully investigate differences in amyloid deposition in this study. Furthermore, there was no association between amyloid deposition, tau deposition, and TSPO signal in either group. In prior PET imaging studies, TSPO signal has been shown to positively correlate with both increasing amyloid and also with tau burden, especially in amyloid positive individuals who are expected to have more advanced disease than those in the present study [78]. Preclinical research suggests that normally functioning TREM2 acts to stop tau propagation, but this only occurs in the presence of significant amyloid pathology [25]. TSPO and tau PET signal have been shown to increase in tandem across the Braak stages, particularly in the presence of a significant amyloid load [9]. We anticipate that when *TREM2* p.R47H carriers develop greater levels of amyloid and tau, the association between these proteins and microglial activation would be disrupted compared to non-carriers. Future research in *TREM2* p.R47H carriers with Alzheimer’s disease could address this but would be challenging due to the rarity of the variant.

Little is known about how the behavioural phenotype differs between *TREM2* p.R47H carriers and non-carriers. In our study, none of the TREM2 p.R47H carriers exhibited parkinsonism or reported psychotic symptoms, unlike previous reports from *TREM2* p.R47H carriers with cognitive impairment [20]. Additionally, there was no difference in scores for depression, anxiety, apathy, fatigue or quality of life between *TREM2* p.R47H carriers and non-carriers, without cognitive impairment diagnosis. However, *TREM2* p.R47H carriers had worse cognitive performance when measured by the ADAS-Cog 13 and DMS, despite none of these participants meeting the criteria for mild cognitive impairment or Alzheimer’s disease. ADAS-Cog 13 is a broad cognitive assessment, that is sensitive for Alzheimer’s disease-related cognitive changes even in early stages of the disease [43]. Word recall and delayed recall are major components of the assessment and assess episodic memory. DMS is a marker for visual episodic memory [79]. These results hint at impaired temporal lobe functions relating to episodic memory in *TREM2* p.R47H carriers, even without overt cognitive impairment. Moreover, the worse visual episodic memory in *TREM2* p.R47H carriers could potentially relate to the higher tau deposition in posterior brain regions (included within the Braak VI region), seen in this study. Further work to evaluate pathology and symptoms linked to this brain region are warranted in *TREM2* p.R47H carriers.

We also demonstrated smaller total brain, white matter and caudate volumes in *TREM2* p.R47H carriers compared to controls. The white matter volume differences are of interest as other *TREM2* variants are associated with leukoencephalopathy [80]. However, no evidence of increased volume of white matter lesions was found in *TREM2* p.R47H carriers, suggesting no marked leukoencephalopathy in this group. Progressive caudate atrophy in mild cognitive impairment and Alzheimer’s disease has previously been described [81]. Patients with Alzheimer’s disease and the *TREM2* p.R47H variant had smaller caudate as well as other frontobasal brain areas [20]. Additionally, in young carriers of the rs143332484 (p.R62H) *TREM2* variant the putamen was found to be smaller. This could suggest that subcortical areas are particularly prone to neurodegeneration in *TREM2* variant carriers or that *TREM2* variants lead to early neurodevelopmental effects in these regions that lead to later life vulnerability to pathology.

There are several limitations of this study which need to be considered. Given the rarity of the *TREM2* p.R47H risk variant, recruitment of only a relatively small number of participants was possible. However, the higher signal to noise ratio of second-generation TSPO tracers can provide sufficient statistical power using relatively modest numbers of participants [29]. None of the *TREM2* p.R47H carriers had a diagnosis of mild cognitive impairment or Alzheimer’s disease. However, considering Alzheimer’s disease pathogenesis initiates years prior to the development of clinical symptoms [67] and that Alzheimer’s disease treatments are increasingly being trialled early in the disease course [82], healthy older high risk adults are an important group of interest in Alzheimer’s disease. The *TREM2* p.R47H carrier and mild cognitive impairment groups had similar levels of Alzheimer’s disease pathology detected on PET. Both groups are at increased risk of Alzheimer’s disease, although the low levels of amyloid and tau pathology detected on PET indicates that conversion to Alzheimer’s disease was not likely imminent in most of the participants. A comparison between these two groups is therefore an important prospective cohort from which future disease outcomes could subsequently be re-evaluated. Other pragmatic decisions included the inclusion of both MAB and HAB TSPO binders, and accepting an age imbalance between groups, which were statistically controlled for. It should be noted that for the key comparison of TSPO signal between the *TREM2* p.R47H carrier group and mild cognitive impairment group, the effect of the 8 years age difference, while needing to be acknowledged, is likely to be marginal [64]. Women have been shown to have higher TSPO signal than men with an alternative TSPO tracer [83]. However, despite the significant sex difference in the TREM p.R47H carrier group with more women compared to the imaging control group, and the older age, the TREM p.R47H carrier group still exhibited lower TSPO signal in early Braak regions, although this was not significant. The microglial activation response is not homogeneous, with microglial phenotype differing in response to the provoking factor, such as amyloid versus tau [84]. TSPO-PET is unable to differentiate between these different types of microglial response, highlighting the need for ongoing preclinical research in this area. It should also be recognised that TSPO is also expressed by astrocytes and endothelial, which may contribute to the signal [85].

Future research should aim to address the role of *TREM2* variants upon microglial activation longitudinally, including later in the disease course after significant accumulation of amyloid and tau, and if feasible with greater numbers of participants. This is especially important as the role of microglia may change across the Alzheimer’s disease course from protective to antagonistic [69]. Moreover, research in younger age groups is important to establish when the brain structural and clinical phenotype changes exhibited in *TREM2* p.R47H carriers first emerge. Altered microglial activation, potentially via TREM2 modulation, is an exciting future target for novel therapeutics in Alzheimer’s disease that is currently undergoing preclinical trials [86, 87].

## Conclusions

We have explored the in vivo impact of the *TREM2* p.R47H mutation in older high disease risk carriers. Carriers of this variant had a suggestion of lower levels of microglial activation in areas of the brain affected by tau pathology early in Alzheimer’s disease pathogenesis. Minor changes in brain structure and the cognitive profile of carriers suggest a different phenotypic profile in *TREM2* p.R47H carriers than non-carriers. Future treatment avenues in Alzheimer’s disease should focus on enhancing the early protective effect of microglia with the aim of stopping the progression of this devastating disease.

### Supplementary Information

**Additional file 1: Table S1.** Effect of TREM2 p.R47H status and influenza vaccine on TSPO binding. **Table S2.** Baseline comparison of regional TSPO binding. **Table S3.** Effect of TREM2 p.R47H status and influenza vaccine on TSPO binding. **Table S4.** Comparison of regional tau binding. **Table S5.** Clinical assessment scores and cognitive battery results. **Figure S1.** Amyloid status and early tau burden.

## Data Availability

The datasets used and analysed during the current study are available from the corresponding author on reasonable request.

## Abbreviations

Aβ: Amyloid-beta
AES: Apathy evaluation score
APOE: Apolipoprotein E
CDR: Clinical dementia rating
DKT: Desikan–Killiany–Tourville
DMS: Delayed matching to sample
DVR: Distribution volume ratio
GDS: Geriatric depression scale
HAB: High-affinity binder
HAI: Haemagglutination inhibition assay
HAM-A: Hamilton Anxiety Rating Scale
KHP-DCR: King’s Health Partners-Dementia Case Register
MAB: Mixed-affinity binder
MCC: Mean choices to correct
MCI: Mild cognitive impairment
MLC: Mean latency to correct
MoCA: Montreal cognitive assessment
MPRAGE: Magnetisation prepared rapid gradient echo
OTS: One Touch Stockings of Cambridge
PAL: Paired associates learning
PRM: Pattern recognition memory
PROTECT: Platform for research online to investigate genetics and cognition in aging
QoL-AD: Quality of life in Alzheimer’s disease
ROI: Region of interest
RVP: Rapid visual information processing
RTI: Reaction time
SD: Standard deviation
SUVR: Standardised uptake value ratio
SSP: Spatial span
SWM: Spatial working memory
TBV: Total brain volume
TE: Total errors
TMT: Trail making task
*TREM2*: Triggering receptor expressed on myeloid cells 2
TSPO: Translocator protein
WM: White matter
