# Supplementary material for: Microglial activation, tau and amyloid deposition in TREM2 p.R47H carriers and mild cognitive impairment patients: a multi-modal/multi-tracer PET/MRI imaging study with influenza vaccine immune challenge
Source: J Neuroinflammation. 2023 Nov 21;20:272. doi: 10.1186/s12974-023-02945-0 (PMC10664604; doi:10.1186/s12974-023-02945-0)

Additional File 1

Table S1 Effect of *TREM2* p.R47H status and influenza vaccine on TSPO binding

	**MCI (*n* = 6)**	***TREM2* p.R47H (*n* = 5)**
**Age** (years) (Median + IQR)	70.7 (66.0 - 77.3)	61.7 (59.5 - 67.0)[a]
**Sex** (Male/Female)	6 / 0	2 / 3
**TSPO genotype** (MAB/HAB)	4 / 2	4 / 1
**APOEε4** (carrier/non-carrier)	4 / 2	3 / 2
**WM - Hypointensity volume** (cm^3) (Median + IQR)	2.8 (1.4 - 7.2)	1.6 (1.2 – 5.5)

HAB = high-affinity binder; IQR = interquartile range; MAB = mixed-affinity binder; MCI = mild cognitive impairment; TSPO = translocator protein; WM = white matter.

APOEε4 carrier refers to the number with ≥1 ε4 allele.

[a]*TREM2* p.R47H carrier significant versus MCI group; $P < 0.05$.

Table S2 Baseline comparison of regional TSPO binding

	**Imaging Controls (n = 8)**	**MCI (n = 8)**	***TREM2* p.R47H (n = 8)**
**Braak I** (mean ± SD)	1.13 ± 0.03	1.12 ± 0.05	1.12 ± 0.03
**Braak II** (mean ± SD)	1.20 ± 0.06	1.23 ± 0.05	1.18 ± 0.05[a]
**Braak III** (mean ± SD)	1.09 ± 0.03	1.11 ± 0.04	1.09 ± 0.02[a]
**Braak IV** (mean ± SD)	1.05 ± 0.01	1.08 ± 0.04	1.08 ± 0.02
**Braak V** (mean ± SD)	1.00 ± 0.02	1.02 ± 0.03	1.04 ± 0.04
**Braak VI** (mean ± SD)	1.02 ± 0.03	1.00 ± 0.05	1.03 ± 0.04

General linear model results for TSPO DVR values based on Braak regions. *TREM2* p.R47H group separately compared to the MCI and imaging control groups. Age and TSPO status as covariates for statistical comparison.

[a]*TREM2* p.R47H carrier significant *versus* MCI group; $P < 0.05$.

DVR = distribution volume ratio; MCI = mild cognitive impairment; SD = standard deviation; TSPO = translocator protein.

Table S3 Effect of *TREM2* p.R47H status and influenza vaccine on TSPO binding

	Beta	CI Lower	CI higher	SE	df	t	sig
**Braak I**							
Vaccine response	0.016	-0.025	0.058	0.018	9.0	0.892	0.40
Group*vaccine response	-0.030	-0.091	0.032	0.027	9.0	-1.098	0.30
**Braak II**							
Vaccine response	0.005	-0.022	0.031	0.01	5.4	0.448	0.67
Group*vaccine response	-0.034	-0.073	0.005	0.015	5.4	-2.195	0.08
**Braak III**							
Vaccine response	-0.001	-0.018	0.016	0.008	12.6	-0.123	0.90
Group*vaccine response	-0.022	-0.046	0.003	0.011	12.6	-1.904	0.08
**Braak IV**							
Vaccine response	0.001	-0.012	0.014	0.006	10.0	0.13	0.90
Group*vaccine response	-0.019	-0.038	0.000	0.009	10.0	-2.171	0.06
**Braak V**							
Vaccine response	0.003	-0.014	0.021	0.008	9.3	0.425	0.68
Group*vaccine response	-0.010	-0.036	0.016	0.012	9.3	-0.878	0.40
**Braak VI**							
Vaccine response	0.003	-0.016	0.022	0.008	9.448	0.382	0.71
Group*vaccine response	-0.007	-0.036	0.021	0.013	9.448	-0.574	0.58

Linear mixed models results for change in TSPO DVR in Braak regions, in response to influenza vaccine challenge and comparison of vaccine response between the *TREM2* p.R47H carrier group relative to the mild cognitive impairment group (Group*vaccine response). Age and TSPO binding status were included as covariates.

CI = confidence interval; df = degrees of freedom; SE = Standard error.

Table S4 Comparison of regional tau binding

	**MCI** (***n*** **= 8)**	***TREM2*** **p.R47H** (***n*** **= 8)**
**Braak I** (mean ± SD)	1.18 ± 0.19	1.14 ± 0.12
**Braak II** (mean ± SD)	1.24 ± 0.17	1.25 ± 0.06
**Braak III** (mean ± SD)	1.12 ± 0.09	1.14 ± 0.05
**Braak IV** (mean ± SD)	1.11 ± 0.08	1.12 ± 0.05
**Braak V** (mean ± SD)	1.02 ± 0.07	1.06 ± 0.03
**Braak VI** (mean ± SD)	0.95 ± 0.06	1.01 ± 0.04[a]

General linear model results for tau SUVR values across Braak regions. Comparison made between mild cognitive impairment (MCI) and *TREM2* p.R47H carrier groups. Age was used as a covariate.

MCI = mild cognitive impairment; SD = standard deviation; SUVR = standardised uptake value.

[a]*TREM2* p.R47H carrier significant *versus* MCI group; $P < 0.05$.

Table S5 Clinical assessment scores and cognitive battery results

Clinical assessments	Clinical Controls		MCI		*TREM2* p.R47H	
**GDS** (median + IQR)	*n* = 29	2.0 (1.0-5.0)	*n* = 11	10.0 (6.0-12.0)	*n* = 12	1.5 (0.0-5.5)[b]
**HAM-A** (median + IQR)	*n* = 28	3.0 (1.0-6.0)	*n* = 11	7.0 (2.0-12.0)	*n* = 12	1.5 (1.0-5.0)[b]
**QoL-AD** (mean ± SD)	*n* = 29	42.0 ± 5.0	*n* = 11	35.2 ± 4.5	*n* = 12	41.2 ± 6.7[b]
**FSS** (median + IQR)	*n* = 27	21.0 (15.0-31.0)	*n* = 11	34.0 (24.0-39.0)	*n* = 11	18.0 (9.0-30.0)[b]
**AES** (mean ± SD)	*n* = 29	63.8 ± 5.5	*n* = 11	66.1 ± 3.4	*n* = 12	66.2 ± 3.4[b]
**Cognitive battery**						
**FAS** (mean ± SD)	*n* = 29	47.9 ± 12.7	*n* = 11	44.1 ± 14.6	*n* = 11	48.5 ± 16.7
**Animal naming** (mean ± SD)	*n* = 29	23.0 ± 5.5	*n* = 11	19.7 ± 3.9	*n* = 11	23.3 ± 7.4
**TMT-A** (median + IQR)	*n* = 29	33.5 (26.9-37.2)	*n* = 11	35.8 (30.3-51.6)	*n* = 11	37.2 (27.0-41.8)
**TMT-B** (median + IQR)	*n* = 29	61.2 (51.4-75.6)	*n* = 11	86.6 (62.8-116.6)	*n* = 11	61.9 (49.6-82.5)
**ADAS-Cog 13** (median + IQR)	*n* = 29	5.3 (3.0-8.0)	*n* = 11	12.0 (10.3-16.3)	*n* = 11	6.7 (4.0-8.3)[a]
**DMS** (mean ± SD)	*n* = 28	88.9 ±6.1	*n* = 11	87.0 ± 7.7	*n* = 11	86.1 ± 6.[a]
**OTS 1st choice** (mean ± SD)	*n* = 25	17.2 ± 4.4	*n* = 10	13.1 ± 3.8	*n* = 11	17.0 ± 2.3
**OTS MCC** (median + IQR)	*n* = 25	1.3 (1.2-1.6)	*n* = 11	1.6 (1.4-2.0)	*n* = 11	1.4 (1.3-1.5)
**OTS MLC** (median + IQR)	*n* = 25	28.2 (20.2-52.5)	*n* = 11	47.0 (40.0-82.0)	*n* = 11	23.4 (15.6-35.3)
**PAL TE** (median + IQR)	*n* = 28	9.5 (6.3-24.0)	*n* = 11	21.0 (13.0-40.0)	*n* = 11	13.0 (8.0-17.0)
**PAL TE 6 shapes** (median + IQR)	*n* = 28	2.5 (0.0-6.0)	*n* = 11	10.0 (3.0-17.0)	n=11	4.0 (2.0-5.0)
**PRM** (median + IQR)	*n* = 28	87.5 (83.3-95.8)	*n* = 11	83.3 (79.2-87.5)	*n* = 11	91.7 (83.3-95.8)
**RTI simple reaction** (mean ± SD)	*n* = 27	322.7 ± 44.6	*n* = 11	370.5 ± 74.4	*n* = 10	328.4 ± 68.3
**RTI five choice** (median + IQR)	*n* = 27	357.0 (307.0-398.0)	*n* = 11	338.5 (328.0-409.5)	*n* = 10	350.0 (298.6-437.6)
**RVP mean latency** (median + IQR)	*n* = 26	457.9 (403.3-592.4)	*n* = 9	459.8 (407.2-540.1)	*n* = 10	459.7 (427.9-493.2)
**SSP span length** (mean ± SD)	*n* = 25	5.6 ± 1.7	*n* = 10	5.0 ± 1.1	*n* = 11	6.0 ± 1.3
**SWM between errors** (mean ± SD)	*n* = 28	37.5 ± 29.2	*n* = 11	47.5 ± 16.9	*n* = 11	36.0 ± 17.1
**SWM strategy** (mean ± SD)	*n* = 28	32.2 ± 8.1	*n* = 11	37.3 ± 5.2	*n* = 11	36.4 ± 4.8

General linear model results for clinical and cognitive assessments values. *TREM2* p.R47H group separately compared to the MCI and clinical control groups. For the cognitive battery age and education were used as covariates. Untransformed values presented.

[a]*TREM2* p.R47H carrier significant *versus* controls; $P < 0.05$.

[b]*TREM2* p.R47H carrier significant *versus* MCI group; $P < 0.05$.

AES = apathy evaluation score; DMS = delayed matching to sample; FFS = fatigue severity scale; GDS = geriatric depression scale; HAM-A = Hamilton Anxiety Rating Scale; IQR = interquartile range; MCC = mean choices to correct; MCI = mild cognitive impairment; MLC = mean latency to correct; OTS = one touch stockings of Cambridge; PAL = paired associates learning; PRM = Pattern Recognition memory; QoL-AD = Quality of life in Alzheimer's disease; RTI = reaction time; RVP = rapid visual information processing; SD = standard deviation; SSP = spatial span; SWM = spatial working memory; TE = total errors; TMT = trail-making task.

Figure S1: Amyloid status and early tau burden. Pooled analysis showing tau Braak I region SUVR values based on amyloid status. High Braak I tau burden is noticeable in amyloid positive participants. SUVR = standardised uptake value ratio.

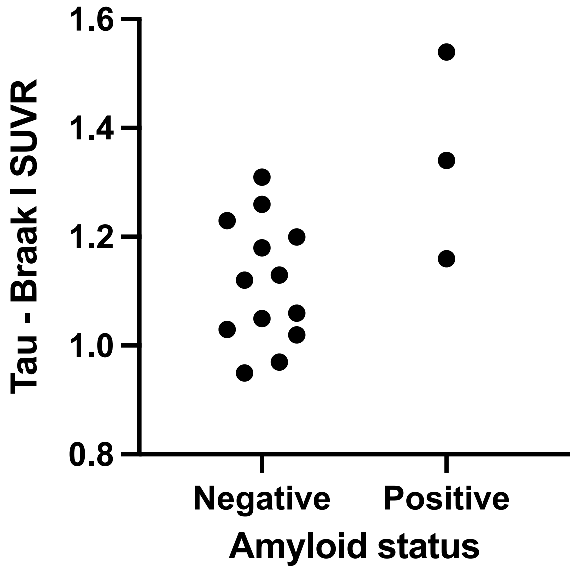

Supplement: Supplementary file 1 — Additional file 1: Table S1. Effect of TREM2 p.R47H status and influenza vaccine on TSPO binding. Table S2. Baseline comparison of regional TSPO binding. Table S3. Effect of TREM2 p.R47H status and influenza vaccine on TSPO binding. Table S4. Comparison of regional tau binding. Table S5. Clinical assessment scores and cognitive battery results. Figure S1. Amyloid status and early tau burden. [file 12974_2023_2945_MOESM1_ESM.pdf]
